# Chlorantraniliprole as a candidate pesticide used in combination with the attracticides for lepidopteran moths

**DOI:** 10.1371/journal.pone.0180255

**Published:** 2017-06-28

**Authors:** Yongqiang Liu, Yu Gao, Gemei Liang, Yanhui Lu

**Affiliations:** State Key Laboratory for Biology of Plant Diseases and Insect Pests, Institute of Plant Protection, Chinese Academy of Agricultural Sciences, Beijing, P. R. China; Institute of Plant Physiology and Ecology Shanghai Institutes for Biological Sciences, CHINA

## Abstract

Methomyl is currently used as a toxicant for the attracticide BioAttract in cotton and vegetables in China. However, methomyl is highly toxic to non-target organisms and a more environmental friendly acceptable alternative is required. Larvae of three lepidopteran insects *Helicoverpa armigera*, *Agrotis ipsilon* and *Spodoptera litura* are important pests of these crops in China. In the present study, the toxicity of 23 commonly used insecticides were tested on *H*. *armigera*, then tested the susceptibility of *A*. *ipsilon* and *S*. *litura* moths to the insecticides which were the most toxic to *H*. *armigera*, and the acute toxicity of the most efficacious insecticides were further investigated under laboratory conditions. Chlorantraniliprole, emamectin benzoate, spinetoram, spinosad and methomyl exhibited high levels of toxicity to *H*. *armigera* moths with a mortality of 86.67%, 91.11%, 73.33%, 57.78% and 80.00%, respectively, during 24 h period at the concentration of 1 mg a.i. L^-1^. Among these five insecticides, *A*. *ipsilon* and *S*. *litura* moths were more sensitive to chlorantraniliprole, emamectin benzoate and methomyl. The lethal time (LT_50_) values of chlorantraniliprole and methomyl were shorter than emamectin benzoate for all three lepidopteran moth species at 1000 mg a.i. L^-1^ compared to concentrations of 500, 100 and 1 mg a.i L^-1^. Chlorantraniliprole was found to have similar levels of toxicity and lethal time on the three lepidopteran moths tested to the standard methomyl, and therefore, can be used as an alternative insecticide to methomyl in the attracticide for controlling these pest species.

## Introduction

Noctuidae is the largest family in the order Lepidoptera. It contains some of the most destructive pests of agricultural crops such as *Helicoverpa armigera*, *Agrotis ipsilon*, and *Spodoptera litura*. In China, larvae of these three species are major pests in many economically-important crops including cotton (*Gossypium* spp.), maize (*Zea mays* Linn.), peanut (*Arachis hypogaea* Linn.), beans (*Glycine max* Linn. Merr.) and vegetables [1]. Larvae of *H*. *armigera* and *S*. *litura* attack leaves, flowers and fruits, and causes extensive damage, while the larvae of *A*. *ipsilon* typically feed on the roots and stems of gramineae and tubers of potato and beet resulting in lodging, wilting and ultimately death of host plants [1]. *H*. *armigera*, *A*. *ipsilon* and *S*. *litura* are all migratory insects [2–4], long-distance migration across different agricultural regions plays a key role in their life history and can result in regional outbreaks [5].

At present, broad-spectrum chemical control targeting the larvae of these three lepidopteran pest species is the most widely practiced management tool. However, the intensive use of insecticides has led to the development of widespread and multiple forms of resistance and severe genitive impacts on non-target species, notably natural enemies and parasitoids. Yang et al. found that 14 populations of *H*. *armigera* from northern China showed high resistance to fenvalerate and phoxim [6]. For *S*. *litura*, high resistance levels against a wide variety of insecticides including profenofos, chlorpyrifos, quinalphos, phoxim, triazophos, methomyl and thiodicarb have been reported from South Asia [7–8]. Therefore, environmentally-friendly control options need to be developed and applied for the management of these lepidopteran pests.

One approach is to specifically target the adults of pest species using odor cues such as pheromones and host kairomones [9]. Such an approach can result in a significant decrease in egg and subsequent larval populations [10]. Host plants emit volatile compounds which attract moths. Recently, the attractants being made of plant volatiles have been developed for trapping both sexes of *H*. *armigera* and other moths [11–13]. Del Socorro et al. (2010) found that methomyl and thiodicarb had high toxicity and were quick acting on *H*. *armigera* moths; methomyl is now the major insecticide used in attracticides for control of *H*. *armigera* moths [13]. In China, a moth attracticide, BioAttract, with methomyl is widely used in the management of lepidopteran pests in cotton, maize, tobacco (*Nicotiana babacum* L.), peanut (*Arachis hypogaea* Linn.) and soybean (*Gycin emax* (L.) Merr.) fields [14–16]. However, methomyl is highly toxic to mammals, fish and aquatic invertebrates, and it has been banned to use on Cruciferous vegetables in China [17]. Therefore, an alternative to methomyl is needed to use in attracticides.

In this study, we evaluated the susceptibility of *H*. *armigera* moths to 23 common insecticides and then tested the susceptibility of *A*. *ipsilon* and *S*. *litura* moths to the insecticides which were the high toxic to *H*. *armigera*. Further, we investigated the acute toxicity of the most efficient insecticides on all three moth pest species.

## Methods

### Ethics statement

No specific permits were required for the collection of *H*. *armigera*, *A*. *ipsilon* and *S*. *litura*, and our study did not involve endangered or protected species.

### Insects

The moths of *H*. *armigera*, *A*. *ipsilon* and *S*. *litura* were captured by a vertical-pointing searchlight trap at the Langfang Experimental Station (39.53° N, 116.70° E), Chinese Academy of Agricultural Sciences (CAAS), in Hebei Province, China. Then, they were stored separately in cages with meshed sides for egg collection. The larvae of *H*. *armigera*, *S*. *litura* and *A*. *ipsilon* were reared using an artificial diet [18–19], at 25±1°C, 60±5% RH with a 14:10 light:dark photoperiod. All moths were provided with a solution of 10% sugar and 2% vitamin complex for nutrition supplement. Moths of third and fourth generations were used for bioassays.

### Insecticides

Technical grade formulations (%, as indicated) of 22 insecticides were tested as follows: methomyl (98%), thiodicarb (95%), chlorantraniliprole (95.3%), flubendiamide (98%), emamectin benzoate (92%), abamectin (97%), spinosad (90%), indoxacarb (94%), fipronil (95%), amitraz (98%), chlorfenapyr (94.5%), phoxim (89%), profenofos (90.7%), beta-cypermethrin (96.5%), deltamethrin (98%), cyhalothrin (95%), fenpropathrin (92%), bifenthrin (97%), fenvalerate (96%), endosulfan (94%), monomehypo (95%) and imidacloprid (95.3%). All these insecticides were provided by the Institute for the Control of Agrochemicals (ICA), the Ministry of Agriculture (MOA), China. Spinetoram suspension concentrate (SC) (50,000 mg a.i. L^-1^) was obtained from Dow AgroSciences, UK.

### Bioassay of *H*. *armigera*, *A*. *ipsilon* and *S*. *litura* moths in the laboratory

A 100 ml stock solution (10,000 mg a.i. L^-1^) of spinetoram was prepared in distilled water, while all other stock solutions (50,000 mg a.i. L^-1^) of insecticides were diluted using dimethyl sulfoxide (DMSO). Each stock solution was further diluted to experimental concentrations with 10% honey solution containing 0.1% Tween-80 (Beijing Chemical Reagent Co. Ltd., Beijing, China). A honey solution containing 1% DMSO and 0.1% Tween-80 was used as a blank control. Cotton balls were soaked with insecticide-honey mixture or honey-only solution, then each cotton ball was placed in flat-bottomed glass tube (22 mm dia., volume 12.1 mL). A single 3-d-old *H*. *armigera* moth was then placed in each tube containing a treated cotton ball. All glass tubes were plugged with a cotton wool and maintained at 25±1°C, 60±5% RH, and a photoperiod of 14:10 h (L:D). The mortality rate of moths in glass tubes was observed after 24h. All bioassay treatments had three replications, and each replicate involved 15 moths of mixed gender.

Bioassays with *A*. *ipsilon* and *S*. *litura* moths were performed in a similar manner to *H*. *armigera* moths, but only with insecticides which demonstrated high toxicity (the mortalities of *H*. *armigera* moths over 50% at the concentration of 1 mg a.i. L^-1^).

### Assessment of median lethal time (LT_50_)

Three concentrations (1000, 100 and 1 mg a.i. L^-1^) of methomyl, chlorantraniliprole and emamectin benzoate containing 10% honey solution and 0.1% Tween-80 were prepared according to their toxicity to three lepidopteran moths, and the honey solution containing 0.1% Tween-80 was used as a control. The 3-d-old moths were used, and starved for 24h before trial. Each treatment was replicated three times, and each replicate included 24 moths. The tethered-flight technique [20], with slight modifications, was used to determine the median lethal time (LT_50_). Tested moths were anesthetized with ether, and scales at the dorsal junction of the thorax and abdomen were gently swept away. Short plastic tethers were glued to the cuticle with 502 adhesive glue (Beijing Chemical Company). A tethered moth was attached to the arm of a flight mill. The time of moth to death was recorded for calculating LT_50_ value of each insecticide at different concentrations. The moth, which has lost the ability to fly, was considered to be dead, because they would be incapable of laying eggs on target crops any more.

### Statistical analysis

Differences in mortality of *H*. *armigera*, *A*. *ipsilon* and *S*. *litura* moths treated by different insecticides were compared by Tukey’s HSD (honestly significant difference) test using SPSS 13.0 software (SPSS Inc., Chicago, IL). The median lethal time, 95% confidence limits (CLs), and slope ± SE were calculated using probit analysis.

## Results

### Toxicity to *H*. *armigera*, *A*. *ipsilon* and *S*. *litura* moths

**Fig 1:**
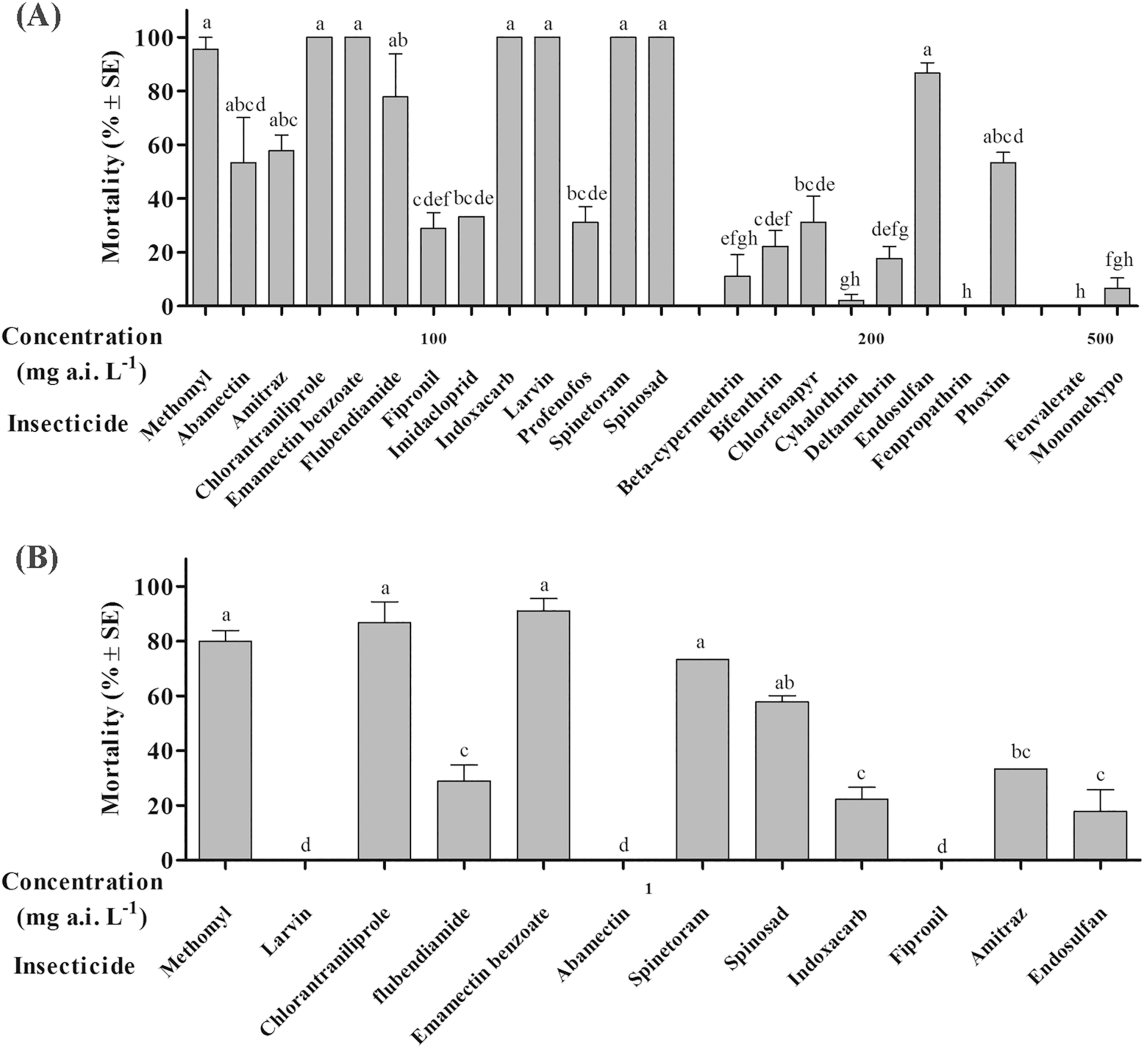
The toxicities of *Helicoverpa armigera* moths after treated by 23 different insecticides following 24 h exposure at the concentration of 100–500 mg a.i. L^-1^ (A) and 1 mg a.i. L^-1^ (B).

Treatments with 23 different insecticides exhibited significant differences in mortality of *H*. *armigera* moths at high concentration (> 100 mg a.i. L^-1^) (*F* = 31.65, df = 22,46, *P*<0.001) compared to mortality in the control groups (<5%). Chlorantraniliprole, emamectin benzoate, indoxacarb, larvin, spinetoram, spinosad, endosulfan, flubendiamide, amitraz, abamectin and phoxim were highly effective with mortalities of 100%, 100%, 100%, 100%, 100%, 100%, 86.67%, 77.78%, 57.78%, 53.33% and 53.33%, respectively, and no significant difference was observed between them and methomyl (Fig 1A).

There were significant differences in the levels of mortality in *H*. *armigera* moths treated with low concentrations (1 mg a.i. L^-1^) of 12 selected insecticides (*F* = 84.59, df = 11,24, *P*<0.001). Chlorantraniliprole, emamectin benzoate, spinetoram and spinosad exhibited the highest levels of toxicity to *H*. *armigera* moths with the mortalities of 86.67%, 91.11%, 73.33% and 57.78% respectively, and no significant difference was observed between them and methomyl (80%). Amitraz, flubendiamide, indoxacarb and endosulfan had relatively low toxicity, with 33.3%, 28.9%, 22.2% and 17.8% morality, respectively. No moths died after being treated with larvin, abamectin and fipronil (Fig 1B).

**Fig 2:**
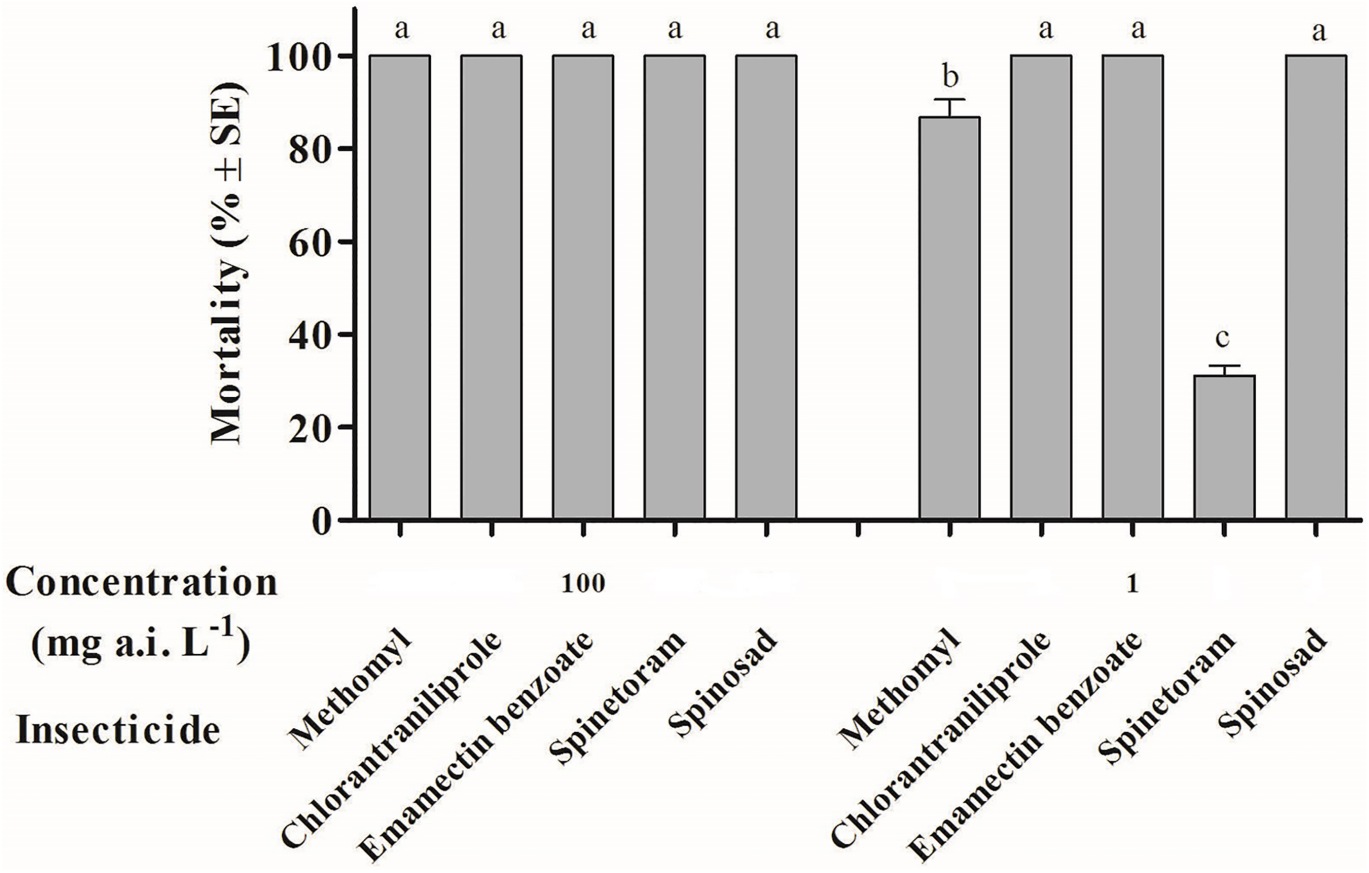
The toxicities of *Agrotis ipsilon* moths after treated by 5 different insecticides following 24 h exposure at the concentration of 100 and 1 mg a.i. L^-1^.

Chlorantraniliprole, emamectin benzoate, spinosad, spinetoram and methomyl had high toxicity to *A*. *ipsilon* moths, and the mortality was 100% after being exposed to a concentration of 100 mg a.i. L^-1^. There were significant differences in mortality of moths treated a low concentration (1 mg a.i. L^-1^) of 5 selected insecticides (*F* = 220.86, df = 4,10, *P* = 0.015). Mortality of *A*. *ipsilon* caused by chlorantraniliprole, emamectin benzoate and spinosad was significantly higher than that of methomyl and spinetoram (Fig 2).

**Fig 3:**
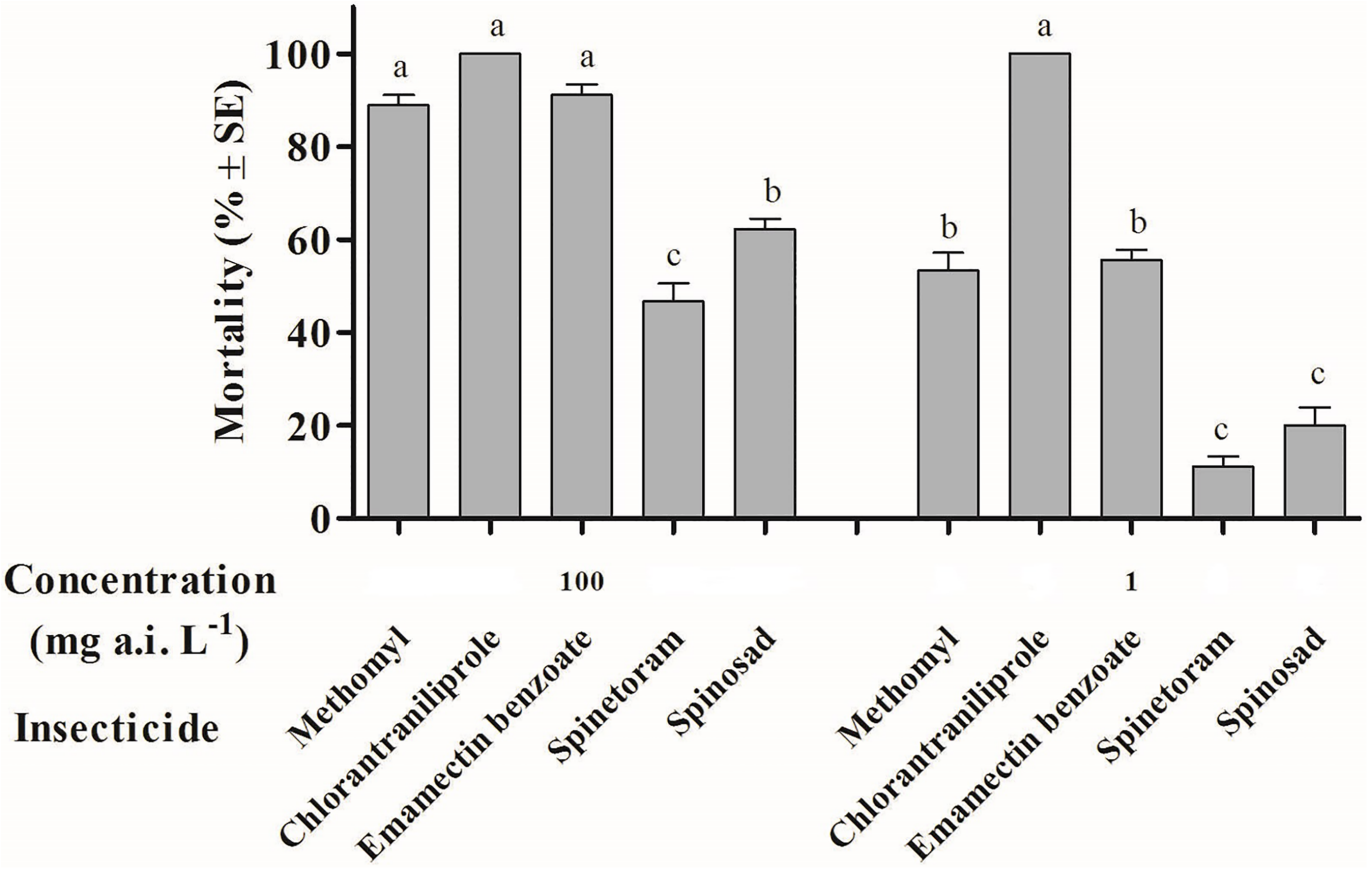
The toxicities of *Spodoptera litura* moths after treated by 5 different insecticides following 24 h exposure at the concentration of 100 and 1 mg a.i. L^-1^.

At a concentration of 100 mg a.i. L^-1^, chlorantraniliprole, emamectin benzoate and methomyl were highly toxic to *S*. *litura* moths with mortalities of 100%, 91.11% and 88.89% respectively, and no significant differences in mortality was observed between them. Moth mortality caused by spinosad and spinetoram was significantly lower than that of chlorantraniliprole, emamectin benzoate and methomyl at a concentration of 100 mg a.i. L^-1^ (*F* = 70.33, df = 4,10, *P*<0.001). There were significant differences in mortality of moths after being treated by a low concentration (1 mg a.i. L^-1^) of 5 selected insecticides (*F* = 86.66, df = 4,10, *P*<0.001). Chlorantraniliprole had the highest toxicity, and caused 100% mortality, followed by emamectin benzoate and methomyl with mortalities of 55.6% and 53.3%, respectively. Spinosad and spinetoram recorded the lowest toxicities, 20.0% and 11.1% respectively (Fig 3).

### Median lethal time (LT_50_) of three lepidopteran moths

At a concentration of 1000 mg a.i L^-1^, the results of the LT_50_ analysis indicated that all three insecticides killed three lepidopteran moths relatively quickly. Methomyl (0.03h, 0.046h and 0.43h) and chlorantraniliprole (0.089h, 0.48h and 0.046h) were faster acting than emamectin benzoate (1.41h, 4.04h and 5.31h) on *H*. *armigera*, *A*. *ipsilon* and *S*. *litura*, respectively. At a concentration of 500 mg a.i L^-1^, chlorantraniliprole (4.86h, 1.97 and 2.09h) exhibited lower LT_50_ than emamectin benzoate (5.82h, 5.95 and 6.93h) and higher LT_50_ than methomyl (0.096, 0.22h and 1.00h) to *H*. *armigera*, *A*. *ipsilon* and *S*. *litura* respectively. At the concentration of 100 mg a.i L^-1^, chlorantraniliprole was the fastest acting insecticide against *S*. *litura* with an LT_50_ of 2.73h. Chlorantraniliprole (8.70h and 2.43h) exhibited a lower LT_50_ than methomyl (0.61h and 0.27h) to *H*. *armigera* and *A*. *ipsilon*. At a concentration of 1 mg a.i L^-1^, all insecticides killed the three lepidopteran moths relatively slowly (>7.5h) (Table 1).

**Table 1:** Lethal time (LT_50_) of three insecticides to the moths of three lepidopteran pests.

Insect	Concentration (mg a.i. L^-1^)	Insecticides	Slope ± SE	LT_50_ (h)	95% Fiducial limits	R^2^(*df*)	*P* values
*H*. *armigera*	1000	Chlorantraniliprole	10.48±1.06	0.089	0.086~0.093	6.81(10)	0.7435
		Emamectin benzoate	7.29±0.73	1.41	1.35~1.48	7.32(13)	0.8850
		Methomyl	4.87±0.47	0.03	0.027~0.032	0.69(10)	0.9999
	500	Chlorantraniliprole	16.78±1.49	4.86	4.76~4.97	8.34(13)	0.8208
		Emamectin benzoate	14.51±1.53	5.82	5.69~5.98	5.50(13)	0.9625
		Methomyl	18.48±1.73	0.096	0.094~0.098	9.41(13)	0.7417
	100	Chlorantraniliprole	2.71±0.32	8.70	7.53~9.99	4.79(10)	0.9048
		Emamectin benzoate	1.92±0.29	7.69	6.17~9.25	4.57(10)	0.9179
		Methomyl	1.51±0.18	0.61	0.47~0.76	4.93(13)	0.9767
	1	Chlorantraniliprole	4.00±0.41	7.56	6.75~8.38	10.11(10)	0.4309
		Emamectin benzoate		>12			
		Methomyl	1.82±0.21	7.53	6.28~9.04	4.11(13)	0.9899
*A*. *ipsilon*	1000	Chlorantraniliprole	6.49±0.62	0.48	0.45~0.50	10.38(13)	0.6624
		Emamectin benzoate	17.20±1.48	4.04	3.95~4.14	7.36(13)	0.8824
		Methomyl	4.62±0.58	0.046	0.041~0.049	3.61(10)	0.9631
	500	Chlorantraniliprole	7.47±0.68	1.97	1.87~2.07	11.68(13)	0.5540
		Emamectin benzoate	19.89±1.80	5.95	5.85~6.06	12.52(13)	0.4853
		Methomyl	11.15±1.01	0.22	0.21~0.23	11.65(13)	0.5566
	100	Chlorantraniliprole	2.17±0.26	2.43	2.02~2.88	6.27(10)	0.7921
		Emamectin benzoate	2.49±0.23	6.79	5.89~7.81	5.92(13)	0.9492
		Methomyl	1.38±0.25	0.27	0.16~0.37	6.35(10)	0.7851
	1	Chlorantraniliprole	1.81±0.17	7.70	6.49~9.27	8.94(16)	0.9158
		Emamectin benzoate	2.10±0.31	17.23	14.25~22.55	5.40(10)	0.8630
		Methomyl	3.21±0.35	13.72	12.13~15.82	11.28(10)	0.3360
*S*. *litura*	1000	Chlorantraniliprole	5.28±0.61	0.046	0.042~0.049	3.45(10)	0.9688
		Emamectin benzoate	7.81±0.75	5.31	5.08~5.57	21.63(13)	0.0613
		Methomyl	5.46±0.62	0.43	0.39~0.47	1.56(10)	0.9987
	500	Chlorantraniliprole	7.90±0.84	2.09	1.99~2.20	5.07(10)	0.8863
		Emamectin benzoate	17.64±1.80	6.93	6.80~7.06	9.43(13)	0.7402
		Methomyl	5.39±0.54	1.00	0.94~1.07	9.16(13)	0.7605
	100	Chlorantraniliprole	1.42±0.12	2.73	2.21~3.33	8.58(19)	0.9799
		Emamectin benzoate	2.24±0.30	9.36	7.90~11.01	6.53(10)	0.7688
		Methomyl	1.63±0.20	4.81	3.79~5.87	6.46(13)	0.9278
	1	Chlorantraniliprole	2.87±0.32	10.13	8.87~11.58	6.32(10)	0.7876
		Emamectin benzoate		>12			
		Methomyl		>12			

## Discussion

Attractiveness of plant volatiles has been widely reported for lepidopteran insects [11–13], and therefore an attracticide, based on combination of synthetic plant volatiles and insecticide, is being used for trapping and controlling lepidopteran moths in Australia, China and other countries [17, 21–22]. This study investigated the toxicity of 23 commonly used insecticides to *H*. *armigera*, then tested the susceptibility of *A*. *ipsilon* and *S*. *litura* moths to the insecticides which were the toxic to *H*. *armigera* moths, and evaluated the acute toxicity of three selected insecticides. The results showed that chlorantraniliprole exhibited high toxicity and fast action to all three pest moth species. Therefore, chlorantraniliprole is a good candidate for controlling lepidopteran moths, and can replace hazardous methomyl in the combination with the application of attracticide products.

Of all the insecticides tested, chlorantraniliprole, emamectin benzoate, spinosad and spinetoram have more toxicity than other insecticides against *H*. *armigera* moths. This result was in accordance with that chlorantraniliprole, emamectin benzoate and spinosad had higher toxicity than indoxacarb and chlorfenapyr on *H*. *armigera* larvae [23]. This study showed that chlorantraniliprole, emamectin benzoate, and spinosad were more toxic than other insecticides tested to *A*. *ipsilon* moths. In addition, chlorantraniliprole displayed high toxicity against *S*. *litura* moths. Xie et al. (2010) studied that it has more efficacy for the control of *S*. *litura* larvae. However, *H*. *armigera* and *S*. *litura* moths appear to be less susceptible to methomyl and spinetoram, respectively, compared with their larvae [23–24]; it may be related to inherent differences in susceptibility between different developmental stages [25].

For the application of an attracticide rapid incapacitation and killing of moths is very important, in order to reduce the opportunity for egg-laying before they died. Though emamectin benzoate showed high toxicity to all three lepidopteran moths, it was not considered to be suitable for use in a moth attracticide because of their relatively slow activity of kill. The LT_50_ value for chlorantraniliprole in three lepidopteran moths tested was found to be low, therefore, the high insecticidal toxicity to target pests make it a good candidate for controlling lepidopteran moths, including *H*. *armigera*, *S*. *litura* and *A*. *ipsilon*.

In order to reduce the lethal time, high concentrations of insecticides are often used in attracticides. For example, the optimum spinosad concentration to cause the fastest mortality rate of *H*. *zea* has been estimated to be approximately 730 mg a.i L^-1^ [26], and the concentration of methomyl used to control *H*. *zea* was estimated to be 105.26 mg a.i L^-1^ [27]. Current experiments used concentrations of 1, 100, 500, 1000 mg a.i. L^-1^ in order to access the effect of concentration on LT_50_, assuming that the amount of fluids imbibed was not influenced by concentration of insecticide. At the concentration of 1,000 mg a.i. L^-1^, chlorantraniliprole had a similar acute toxicity to methomyl. Previous studies have showed that sublethal concentrations of spinosad and emamectin benzoate significantly reduced oviposition of *H*. *zea*. Also, the sublethal concentrations of emamectin benzoate significantly impacted the survival of *H*. *zea* larvae [26, 28]. Hence, the sublethal effects of chlorantraniliprole against lepidopteran moths needs to be more further studied, for reduce the dosage in attracticide, although the use of such concentrations are risk able for following increasing rate of insecticides its can developed resistance power in target species.

Chlorantraniliprole, is an anthranilic diamide insecticide, which specifically targets insect ryanodine receptors (RyRs) that are critical for muscle contraction in insects [29–30] and this specificity means that the insecticide has very low mammalian toxicity. Activation of the ryanodine receptors in insects affects uncontrolled release of calcium from internal stores in the sarcoplasmic reticulum, causing unregulated release of internal calcium in the cell and leading to feeding cessation, lethargy, muscle paralysis, and ultimately death of the insect [29]. Brugger et al. (2010) reported that chlorantraniliprole had selectivity to the beneficial parasitoid wasps *Aphidius rhopalosiphi*, *Trichogramma dendrolimi*, *Trichogramma chilonis*, *Trichogramma pretiosum*, *Aphelinus mali*, *Dolichogenidea tasmanica* and *Diadegma semiclausum* [31]. This insecticide was also minimally toxic to larvae and adults of the predators *Harmonia axyridis* and *Chrysoperla sinica* [32]. Therefore, considering the safety of parasitoids and natural enemies, chlorantraniliprole is an ideal toxicant for use with attracticides such as BioAttract.
